# Mapping of serological testing and SARS-CoV-2 seroprevalence studies performed in 20 European countries, March-June 2020

**DOI:** 10.7189/jogh.11.05014

**Published:** 2021-07-31

**Authors:** Laura Bubba, Peter Simmonds, Thea K Fischer, Heli Harvala

**Affiliations:** 1 Department of Biomedical Science for Health, University of Milan, Milan, Italy; 2 Nuffield Department of Medicine, University of Oxford, Oxford, UK; 3 Department of Clinical Research, University hospital of Nordsjaelland, Hilleroed, Denmark; 4 Department of Public Health, University of Copenhagen, Copenhagen, Denmark; 5 Microbiology Services, NHS Blood and Transplant, London, UK; 6 Division of Infection and Immunity, University College London, London, UK

## Abstract

**Background:**

The SARS-CoV-2 pandemic spread across Europe from February 2020. While robust SARS-CoV-2 serological assays were quickly developed, only limited information on applied serological testing is available. We describe the extent and nature of SARS-CoV-2 serological testing used in Europe and assess the links between epidemiology, mitigation strategies applied and seroprevalence.

**Methods:**

An online questionnaire on SARS-CoV-2 serology was sent to the European Society of Clinical Virology and European Non-Polio Enterovirus Network members in September 2020. Data were analysed by comparing mitigation approaches, serological methods and seroprevalance studies performed.

**Results:**

About 100 000 laboratory confirmed cases identified between March and June 2020 were reported by 36 participating laboratories from 20 countries. All responders experienced mitigation strategies including lockdowns and other closures. All except one participant had introduced serological testing; most had validated their assays (n = 29), but some had had difficulties in obtaining reference material. Most used commercial assays (n = 35), measuring IgG response against the spike antigen. Serology was used primarily for diagnostic purposes (n = 22) but also for convalescent plasma (n = 13) and research studies (n = 30). Seroprevalence studies targeted mainly health care workers (n = 20; seroprevalance 5% to 22%) and general population (n = 16; seroprevalance 0.88% to 5.6%). Basic demographic and clinical information were collected by most laboratories (n = 28), whereas data on long-term outcomes were rarely collected.

**Conclusions:**

This is first study gathering systematic information on serological testing approaches implemented during the first pandemic wave in Europe.

Infection with severe acute respiratory syndrome coronavirus 2 (SARS-CoV-2) was first reported in Wuhan, China, in December 2019 [1]. It soon escalated into a pandemic which has to date as of 8 April 2021 affected more than 27 million people and been associated with more than 625 000 deaths in the European region [1]. SARS-CoV-2 associated disease termed as COVID-19 ranges from asymptomatic to mild upper respiratory track and gastrointestinal symptoms, to severe pneumonia, thrombosis, multi-organ failure and death [2].

During the first pandemic wave in Europe, comprehensive mitigation strategies were implemented in many European countries in order to limit the spread of the novel SARS-CoV-2 and to protect health care systems from overwhelming numbers of very ill patients. These measures included national lockdowns and social isolation, enhanced border control and even closure of borders. At the same time, huge efforts were required from diagnostic virology laboratories and public health agencies to evaluate and introduce new molecular and serological methods for SARS-CoV-2 diagnostics in order to respond to the pandemic and provide on-going scientific support for the mitigation strategies introduced. For these reasons, population-based estimates for SARS-CoV-2 seroprevalence were, and still are, in high demand. Review of published data highlighted a generally low SARS-CoV-2 seroprevalence in Europe [3]. Nationwide seropositivity ranged from 2.5% in Italy [4] and 5% in Spain [5], despite the similar numbers of SARS-CoV-2 infections reported during the first wave [6]. However, seropositivity numbers should not be compared without adjusting for test sensitivity and specificity, highly dependent on the chosen target antigen and assay used. Furthermore, substantial variability in SARS-CoV-2 seropositivity was demonstrated in Spain. Highest seroprevalence rates exceeding 10% were measured in the areas with high prevalence of SARS-CoV-2 infection (ie, Madrid area) and among health care workers with likely greater exposure to the virus.

Serological tests are used for many purposes including estimating population exposures, retrospective diagnostics of SARS-CoV-2 infections, and identification of convalescent plasma donations containing high levels of SARS-CoV-2 antibodies [7].

A large number of serological assays have been released into European market since the first reports of SARS-CoV-2 infection in Wuhan. Commercially available serological assays can detect IgG, IgM or IgA alone or combination of all antibodies (total antibody). Existing assays target antibodies to the nucleocapsid and/or spike protein, sometimes including only the receptor-binding domain (RBD) part. Although fast development of commercial serological assays has been vital to our pandemic response, their performance including sensitivity and specificity has remained suboptimal at times [8]. However, no information is available, in our knowledge, about the European diagnostic laboratory approach to serology, including how commonly and which serological methods are used, and how these were validated. Furthermore, limited data are published on the quality of serological data collected and their completeness, which is needed in order to better understand the antibody response acquired as a result of SARS-CoV-2 infection.

We have evaluated the extent of serological SARS-CoV-2 testing and methods used in Europe so far and the quality and quantity of serological data collected during the first wave of pandemic. Mitigation and quarantine strategies applied have been also investigated in order to describe whether they affected the number of COVID-19 cases. These will provide essential European-wide baseline information which can be used to inform future policies and targeted public health strategies during the future pandemic waves and to evaluate vaccine immunization response.

## METHODS

### Data collection

A link to the online questionnaire was sent to all members of the European Society for Clinical Virology (ESCV) and European Non-Polio Enterovirus Network (ENPEN) in order to reach a good selection of clinical and public health virology laboratories on the European territory. One reminder letter was sent. Data were collected using the EU-survey platform. Questionnaire was opened 23rd September and closed 12th October.

**Figure 1:**
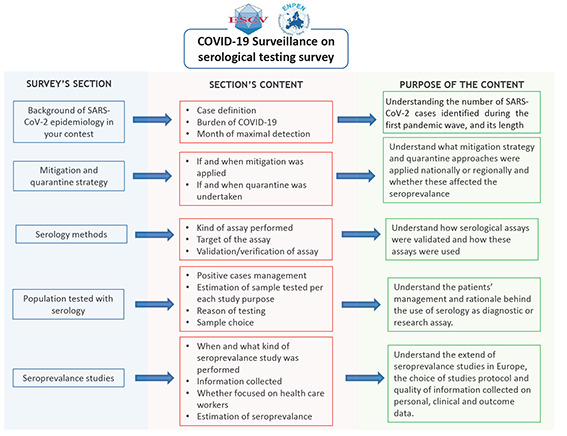
Survey structure divided by the five sections of the survey, their specific content and the purpose of the section.

Basic participant information was collected; this included whether their laboratory was performing diagnostic SARS-CoV-2 services and whether it was linked to hospital, university and/or national public health institution. The survey focused on the epidemiology of SARS-CoV-2 infections in the respondent’s institution or country, and hence the number of SARS-CoV-2 infections diagnosed, and population size covered by that institution were captured (**Figure 1**). Data on mitigation and quarantine strategies applied were also collected. Details of serological testing methods applied in respondent’s institution or country, including their details of their validation, use in diagnostic and different seroprevalence studies, was also collected. Data on serological testing performed was collected, including the estimated number of samples tested.

### Data analyses

A descriptive analysis was undertaken by participant laboratory and country. The geographical distribution of laboratories by their main function in Europe were mapped.

Participant laboratories were asked to report the COVID-19 case definition in use during the study period.

Responders also reported, information on the month when the highest number of confirmed SARS-CoV-2 cases, and this was compared with the timing of lockdown strategy applied. The estimated number of COVID-19 cases was compared to the length of mitigation strategies applied, in order to demonstrate whether to highest number of cases corresponded to the longest period of restrictions applied.

Serological methods reported by participant laboratory were divided into virus neutralisation, commercial and in-house assays. In addition, we collected and analysed the data on targets (ie, immunoglobulin class or antigenic target) and we recorded if the laboratories has performed the validation or verification of their serological assay, and if so, using how many samples. Serological testing was categorised into clinical diagnostics, testing of convalescent plasma donor to determine which donations contained high enough SARS-CoV-2 antibody levels to be used as a treatment for COVID-19 and seroprevalence studies (ie, serology was used to drive or support studies to determine the susceptibility of the population calculating the seroprevalance in a population).

For seroprevalance studies, we collected and analysed data on study population, design and size of the study.

Laboratories performing seroprevalance studies were asked to estimate the seroprevalance as the proportion of SARS-CoV-2 seropositives by the number of inhabitants, and the study population size when possible. The proportions reported were compared, analysing whether any significant differences were observed with χ^2^ test, considering a *P* < 0.05 as statistically significant.

## RESULTS

**Figure 2:**
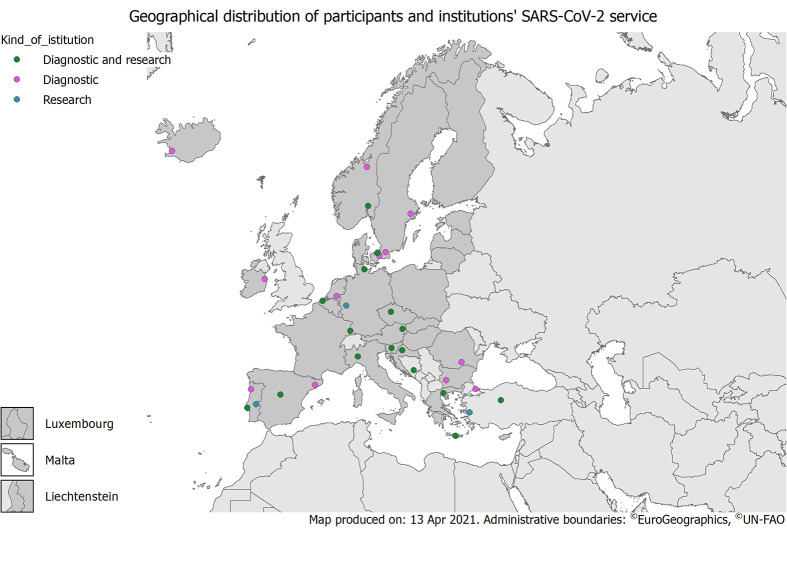
Map of the 36 collaborating laboratories divided by purpose of laboratory work: 1) “diagnostic and research” (in green) include all laboratories belonging to university hospitals or hospitals including a research section; 2) “diagnostic” (in violet) include all hospital laboratories, private laboratories and national/regional reference centres that operate also as diagnostic laboratory; 3) “research” (in blue) consist of all university sites and laboratory mainly dedicated to research projects.

A total of 36 participant laboratories from 20 countries responded to this survey (**Table 1****,** **Figure 2**). Portugal was represented with 6 participant laboratories, followed by Germany, Spain and Turkey which contributed with 3 participating laboratories and Denmark, Greece, Norway, Slovenia and Sweden with two participating laboratories each. Austria, Belgium, Bosnia and Herzegovina, Bulgaria, Croatia, Czech Republic, Iceland, Ireland, Italy, the Netherlands and Romania participated with one laboratory each. Eight countries reported the population covered from their laboratories and/or the number of beds accounted in their hospitals. Reported population size ranged from 700 bed hospital in Istanbul to 1616 beds in Freiburg, covering approximatively from 500 000 and >1 000 000 inhabitants (**Table 1**).

**Table 1:** List of kind of institutions, estimate number of COVID-19 confirmed cases identified during the study period, population served/number of hospital beds and mitigation and quarantine schemes applied from March to June 2020 by participant laboratories

Country	City (in order of submission)	Institution	Purpose laboratory activity	Population	Total number of cases reported (estimate)	Peak month	Lockdowns implementation				School, office and restaurants closure				Quarantine scheme			
**March**	**April**	**May**	**June**	**March**	**April**	**May**	**June**	**Confirmed close contact**	**Suspected close contact**	**Travel in EU countries**	Travel extra EU countries
**Austria**	Vienna	Hospital and University microbiology laboratory	Diagnostic and research	Not reported	101-1000	March	*	*	**	**	¶	¶	¶	**	<14 d, depending on swab results	<14 d, depending on swab results	<14 d, 14 d	<14 d, 14 d
**Belgium**	Bruges	Hospital microbiology laboratory	Diagnostic	Not reported	1000-5000	April	*	*	**	‡	**	**	**	¶	14 d, depending on swab results		14 d	14 d
**Bosnia and Herzigovina**	Sarajevo	Hospital and University microbiology laboratory	Diagnostic and research	1 331 310 people; 1464 beds	1000-5000	June	-	*	*	**	**	¶	¶	§	14 d			
**Bulgaria**	Sofia	National public health institute	Diagnostic and research	Not reported	<10	Not reported	*	*	*	*	¶	¶	¶	¶	>14 d, depending swab/sero results	14 d, depending swab/sero results	14 d, depending swab/sero results	14 d, depending swab/sero results
**Croatia**	Zagreb	National public health institute	Diagnostic and research	Not reported	1000-5000	April	*	*	*	‡	¶	¶	¶	§	14 d depending on swab results		14 d	14 d
**Czech Republic**	Prague	National public health institute	Diagnostic and research	Not reported	101-1000	April	*	*	*	-	¶	¶	¶	**	<14 d	Depending on swab results	14 d	14 d
**Denmark**	Copenhagen	Hospital microbiology laboratory	Diagnostic	>1 000 000 people	1000-5000	April	*	*	*	‡	¶	¶	¶	¶	14 d	14 d	14 d	14 d
	Copenhagen and Hilleroed	National public health institute; University laboratory or research unit	Diagnostic and research	Not reported	>10 000	March	*	*	*	*	¶	¶	¶	‖	14 d	14 d, depending on swab results	14 d	14 d
**Germany**	Kiel	Regional public health institute; Hospital and University microbiology laboratory	Diagnostic and research	Not reported	10-100	March	*	*	**	**	¶	¶	§	**	14 d	Depending on swab results	14 d	14 d
	Freiburg	Hospital and University microbiology laboratory	Diagnostic and research	1616 beds	101-1000	April	*	*	**	**	**	**	**		14 d, depending on swab results	Depending on swab results		
	Bonn	University microbiology laboratory	Research	Not reported	101-1000	March	*	*	**	**	¶	¶	**		14 d		14 d	14 d
**Greece**	Thessaloniki	University microbiology laboratory	Diagnostic and research	Not reported	101-1000	April	*	*	*	‡	**	¶	¶	¶	14 d	14 d	14 d	14 d
	Crete	Regional public health institute	Diagnostic and research	Not reported	10-100	June	*	*	*	**	**	**	**	**	14 d	14 d	14 d	14 d
**Iceland**	Reykjavík	Hospital microbiology laboratory	Diagnostic	Not reported	1000-5000	March	**	**	**	**	‖	‖	**	**	14 d	14 d	14 d	14 d
**Ireland**	Dublin	Hospital microbiology laboratory	Diagnostic	Not reported	1000-5000	April	*	*	*	‡	**	**	**	**	14 d	14 d	14 d	14 d
**Italy**	Pavia	Regional public health institute; University laboratory or research unit	Diagnostic and research	Not reported	5000-10 000	March	*	*	*	**	¶	¶	¶	§	14 d, depending swab/sero results	14 d depending swab/sero results	14 d, depending swab/sero results	14 d, depending swab/sero results
**The Netherlands**	Tilburg	Hospital microbiology laboratory	Diagnostic	5 000 000 people; 1000 beds	1000-5000	April	*	*	**	**	¶	¶	**	**	14 d, depending swab/sero results	14 d, depending swab/sero results	<14 d	<14 d
**Norway**	Oslo	Hospital and University microbiology laboratory	Diagnostic and research	Not reported	1000-5000	March	*	*	**	**	¶	¶	**	**	14 d	14 d, depending on swab results	<14 d	<14 d
	Trondheim	Hospital microbiology laboratory	Diagnostic	470.000 people	101-1000	March	*	*	**	**	¶	¶	**	**	14 d	14 d	14 d	14 d
**Portugal**	Porto	University microbiology laboratory	Research	Not reported	Not reported	Not reported	*	*	*	**	¶	¶	¶	**	14 d	14 d		
	Mora	University laboratory or research unit; Community Pharmacy	Diagnostic and research	Not reported	10-100	Not reported	Not reported								14 d	14 d	Depending on swab results	Depending on swab results
	Lisbon 1	National public health institute; Hospital and University microbiology laboratory	Diagnostic and research	Not reported	101-1000	April	*	**	**	**	¶	¶	**	**	14 d	14 d	Depending on swab results	Depending on swab results
	Lisbon 2	National Institute of Health	Diagnostic and research	Not reported	>10 000	May	*	*	*	**	¶	¶	¶	**	14 d	14 d	Depending on swab results	Depending on swab results
	Porto	National public health institute	Diagnostic and research	Not reported	10-100	April	*	*	**	**	¶	¶	¶	§	14 d			
	Penafiel	Private Laboratory Clinical Analysis	Diagnostic	Not reported	101-1000	June	*	*	*	**	¶	¶	¶	**	14 d	14 d		
**Romania**	Bucharest	Hospital microbiology laboratory	Diagnostic	Not reported	5000-10 000	June	*	*	**	**	¶	¶	¶	¶	14 d	Depending on swab results	14 d	14 d
**Slovenia**	Ljubljana 1	Diagnostic laboratory at the National public health institute	Diagnostic and research	Not reported	10-100	April	*	*	**	**	¶	¶	**	**	14 d		14 d	14 d
	Ljubljana 2	Academic diagnostic microbiology laboratory	Diagnostic and research	Not reported	101-1000	March	*	*	**	**	¶	¶	**	**	<14 d		<14 d	<14 d
**Spain**	Barcelona 1	Hospital microbiology laboratory	Diagnostic	Not reported	1000-5000	March	*	*	*	**	¶	¶	¶	§	14 d	14 d		
	Barcelona 2	Hospital microbiology laboratory	Diagnostic	450 000 people; 1200 beds	>10 000	March	*	*	‡	‡	¶	¶	¶	§	14 d	14 d		
	Madrid	National public health institute	Diagnostic and research	Not reported	Not reported	Not reported	Not reported								Not reported			
**Sweden**	Stockholm	Hospital microbiology laboratory	Diagnostic	Not reported	>10 000	April	Recommendations on social distancing ie, working from home, max allowed gatherings 50 people, restricted access to caring facilities								Not reported			
	Lund	Hospital microbiology laboratory	Diagnostic	Not reported	1000-5000	June									Not reported			
**Turkey**	Istanbul	Hospital and University microbiology laboratory	Diagnostic and research	700 beds	5000-10 000	April	*	*	*	**	¶	¶	¶	**	14 d	Depending on swab results	14 d	14 d
	Izmir	University microbiology laboratory	Research	1809 beds	101-1000	April	**	*	*	*	¶	¶	¶	¶	14 d	14 d	14 d	14 d
	Ankara	University microbiology laboratory	Diagnostic	1040 beds	1000-5000	April	**	†	†	**	**	¶	¶	¶	14 d, depending on swab results	14 d	14 d	14 d

d – day

*National, Regional and Local lockdowns.

†Regional and local lockdowns.

‡Local lockdowns only.

§School and office closure.

‖Restaurant closure.

¶School, office and restaurant closure.

**No lockdown and/or closures applied.

The majority of laboratories participating in the survey were involved in both diagnostic work and research (20/36, 55%); these included university hospitals (5/20), national (9/20) or regional (3/20) public health institutes and university microbiological laboratories (3/20). The remaining were diagnostic hospital laboratories (11/16), private laboratory (1/16) or university departments (4/16) (**Table 1**).

### Case-definition

Thirty-three out of 36 participating laboratories reported use of the COVID-19 case definition during the study period: in most cases viral RNA detection in nasal-pharyngeal specimen or secretions taken from the respiratory tract was considered sufficient to confirm a case (70%, 23/33), whereas the remaining responders defined a confirmed COVID-19 case only when clinical symptoms consistent with COVID-19 were reported in addition to molecular RNA detection (28%, 9/33). Case definition was not applicable for three responders (ie, private laboratories) and one participant did not describe a case definition. Notably, as the case definition might have changed for some locations during the first wave of the pandemic, coinciding with the study period, the most recent case definition was recorded.

### Case numbers

Around 100 000 laboratory confirmed SARS-CoV-2 infections identified between 1st March and 31st June 2020 were reported by the 34 participant laboratories. However, it is important to note that this was an estimate only and the number of reported cases ranged from less than 10 reported by Bulgaria to more than 10 000 cases reported by participant laboratories in Denmark, Portugal, Spain and Sweden (**Table 1**). The highest number of infections were recorded in March by 11 laboratories from 6 countries and in April by 15 laboratories from 10 countries (**Table 1**). Only one participating laboratory recorded a peak number of cases in May (Portugal) and five in June. No such data were obtained from 4 laboratories.

### Mitigation and quarantine strategies

Mitigation and quarantine strategies were reported by 33 participants. Sweden (reported by both participating laboratories) was the only country which did not apply any mitigation or lockdown during the first pandemic wave, but were instead recommending people to keep social distancing, avoid gathering with more than 50 people and restricting the access to care homes (**Table 1**). 30 out of the remaining 32 participant laboratories reported a national lockdown undertaken as mitigation strategy, with varying length. For 26 participating laboratories, it was possible to compare the timing of the peak of COVID-19 case detection and implementation of mitigation measures. It was observed that the peak number of cases were recorded one month after the introduction of mitigation measures by 42% of participant laboratories (11/26), by 39% (10/26) during the first month of lockdown, by 15% (4/26) the month after the end of lockdown and by 4% (1/26) during the last month of these actions (**Table 1**). Severity of mitigation strategies, such as the length of national lockdown was not always proportional to the number of cases reported. For instance, Bulgaria reported less than 10 cases, whereas over 10 000 cases were reported by the Danish participants. Despite the difference in reported number of SARS-CoV-2 infections, both countries applied four months of lockdown (**Table 1**). Six countries including Belgium, Croatia, Greece, Ireland, and Spain reported the use of local lockdowns in spite of the national lockdown in order to control the increasing numbers of SARS-CoV-2 infections in the affected areas (**Table 1**).

Most laboratories reported the use of 14-day isolation period for the close contacts of confirmed SARS-CoV-2 case (29/33) and some of them applied these also for the contacts of suspected SARS-CoV-2 case (20/33). All laboratories placed contacts into 14-day quarantine while waiting for the PCR result and that was also applied for foreign travel (17/33; **Table 1**).

### Serological assays used

All except one laboratory had introduced at least one SARS-CoV-2 serological assay for routine work during the first pandemic wave (n = 35), while the remaining laboratory was planning to introduce such a test. All of them had introduced commercial assay for SARS-CoV-2 serology, and one focused on live virus neutralisation testing (Kiel; **Table 2**). Among the commercial assays performed, Euroimmun ELISA was the most common (17 participating laboratories from 36), followed by Roche (n = 12), Abbott (n = 10), Diasorin (n = 7) and Wantai (n = 7; **Table 2**). Three laboratories used in-house assays and eight were performing neutralising antibody testing in addition to the commercial methods. These included neutralisation testing using live virus (n = 7; three laboratories in Germany, one in Croatia, Italy and Norway), pseudotype (Ireland) or other surrogate neutralising antibody testing (Spain, **Table 2**).

**Table 2:** List of methods performed to detect SARS-CoV-2 antibodies and/or antigens and commercial assay in use during the study period, by participant laboratories, from March to June 2020

Country	City	Antibody detection				Antigens			Live virus neutralisation assay	Commercial assay details							In-house assays
**IgG**	**IgM**	**IgA**	**Total Ig**	**Spike**	**RBD**	**N**	**Euroimmun**	**Roche**	**Abbott**	**Diasorin**	**Wantai**	**NovaTec**	**Other**
**Austria**	**Vienna**	×	×	×	×	×	×	×	×	×	×			×		×	×
**Belgium**	**Bruges**	×	×			×		×		×		×			×	×	
**Bosnia and Herzegovina**	**Sarajevo**	×	×				×									×	
**Bulgaria**	**Sofia**	×	×	×			×			×							
**Croatia**	**Zagreb**	×	×	×		×		×	×	×		×			×	×	
**Czech Republic**	**Prague**	×	×	×			×	×		×							
**Denmark**	**Copenhagen**	×	×													×	
	**Copenhagen and Hilleroed**	×	×											×			
**Germany**	**Kiel**								×[9]	No commercial kit in use							
	**Freiburg**	×							×	×							×
	**Bonn**	×	×	×	×			×	×	×	×	×		×		×	
**Greece**	**Thessaloniki**	×	×	×	×										×	×	
	**Crete**	Not reported								×							
**Iceland**	**Reykjavík**	Not reported									×						
**Ireland**	**Dublin**	×	×						×*			×	×	×			
**Italy**	**Pavia**				×	×		×	×				×				
**The Netherlands**	**Tilburg**				×		×							×			
**Norway**	**Oslo**	×	×	×	×	×	×			×	×		×			×	
	**Trondheim**				×		×	×	×		×			×			
**Portugal**	**Porto**	×	×									×					
	**Mora**	×	×									×					
	**Lisbon 1**	×	×								×		×				
	**Lisbon 2**	×	×	×	×	×	×			×				×			
	**Porto**	×	×													×	
	**Penafiel**	×										×					
**Romania**	**Bucharest**	×	×													×	
**Slovenia**	**Ljubljana 1**	×	×	×		×		×		×						×	
	**Ljubljana 2**	×	×	×	×	×	×	×	×	×	×						
**Spain**	**Barcelona 1**	×	×									×			×		
	**Barcelona 2**	×		×	×	×		×		×	×		×				
	**Madrid**	×		×		×	×		×*	×	×	×					
**Sweden**	**Stockholm**	×				×		×					×			×	×
	**Lund**	×				×					×		×			×	
**Turkey**	**Istanbul**	×	×	×	×					×	×	×				×	
	**Izmir**	×		×	×	×		×		×	×						
	**Ankara**	×				×				×							
**Total**	**37**	**30**	**23**	**12**	**11**	**12**	**7**	**10**	**8**	**17**	**12**	**10**	**7**	**7**	**4**	**14**	**3**

*Pseudotype virus neutralisation assay [10].

Each laboratory using commercial or in-house serological assays reported availability to measure IgG response alone (3/36 laboratories) or together with other immunoglobulins (27/36); further testing included IgM (23/36) or IgA antibodies (12/36). Assays targeted most often the spike protein (S, n = 12) but also nucleoprotein (N, n = 10) or receptor binding domain of spike protein (RBD, n = 8). Only two laboratory reported availability to measure antibody responses against all three target proteins (Austria and one Slovenian laboratory, **Table 2**).

### Assay validation

29 out of 36 laboratories reported that their method had been validated before introduction into clinical use. Among the remaining seven laboratories, two reported that the internal validation performed by their testing system was sufficient enough for them and hence no further assessment was required. Two laboratories could not identify sufficient control material for validation, one relied on published validation data on the methods selected for use and one decided to introduce the emergency test for convalescent plasma donor screening without validation. The number of known positive and negative samples included in validation work varied from one sample each to over 500 samples. Four laboratories (14%) had included fewer than 15 samples in their validation panel.

Most laboratories reported the use of serum samples only (20 from 34 responses), whereas 12 laboratories also accepted plasma, one saliva and one plasma and saliva in addition to serum. Only one laboratory was limited to using plasma or capillary blood only.

### Application of serological testing

Participating laboratories were asked to report the purpose of serological testing in use (see section *Serological assays used* above). In particular 30/36 laboratories declared to use SARS-CoV-2 serological testing as support for seroprevalance studies (**Table 3**), 22 laboratories as a diagnostic service, 13 laboratories performed serological testing to support collection of the convalescent plasma and 8 as part of larger studies, including also other methodologies of detection (**Table 4**).

**Table 3:** Focus on the seroprevalance studies conducted during the study period by participant laboratories: overview of reported study designs, estimation of seroprevalence calculated and estimation of the number of samples included into each study (when available) and/or overall reported in the last column.

Country	City	Seroprevalance studies										
**School or other workplace**		**Hospital or GP associated**		**Blood donors**		**Health care workers (HCW)**		**General population**		**Estimated number of samples included**
**Study design**	**Estimated seroprevalence**	**Study design**	**Estimated seroprevalence**	**Study design**	**Estimated seroprevalence**	**Study design**	**Estimated seroprevalence**	**Study design**	**Estimated seroprevalence**	
**Austria**	Vienna	Descriptive cross-sectional	Manuscript submitted	Descriptive cross-sectional	Manuscript submitted	Cohort; Descriptive cross-sectional	Study ongoing	Cohort; Descriptive cross-sectional	Manuscript submitted	Cohort; Descriptive cross-sectional	Manuscript submitted	>5,000
**Belgium**	Bruges			Cohort of dialysis patients	2.5% [11]			Descriptive cross-sectional	11%	Descriptive cross-sectional	5.6%	
**Bulgaria**	Sofia	Unknown						Unknown				
**Croatia**	Zagreb			Targeted				Convenient sampling		Convenient sampling		1.05
**Czech Republic**	Prague	Descriptive cross-sectional						Unknown				460
**Denmark**	Copenhagen and Hilleroed							Cohort; Descriptive cross-sectional	5%	Descriptive cross-sectional	2.2%	30 000 HCW ~ 18 000 general population
**Germany**	Kiel									Convenient sampling		
	Freiburg	Unknown	Not reported					Unknown	Not reported			1,000
	Bonn	Unknown	Not reported					Unknown	Not reported			>4000
**Greece**	Crete					Cohort study	Not reported	Cohort study	Not reported	Cohort study	Not reported	2,000
**Ireland**	Dublin									SCOPI study [12]		1,733
**Italy**	Pavia	Descriptive cross-sectional study	Not reported	Unknown	Not reported	Unknown	Not reported	Unknown	Not reported	Unknown	Not reported	5,000
**The Netherlands**	Tilburg							Cohort study	22%			~ 140
**Norway**	Oslo	Unknown				Unknown		Unknown		Unknown		~ 4500
	Trondheim	Unknown				Unknown		Unknown		Unknown		400
**Portugal**	Porto					Cohort; Descriptive cross-sectional						525
	Mora	Unknown										250
	Lisbon 2									Convenient sampling	Seroprevalence [2.9% IC95(2.0%-4.2%)] crude; [1.7 IC95(0.0 – 3.3)] adjusted for test sensitivity	2,300
	Porto							Unknown				60
	Penafiel									Convenient sampling	2.7% (only igg was evaluated)	2724
**Romania**	Bucharest							Cohort study				Not know
**Slovenia**	Ljubljana 1							Descriptive cross-sectional	Still ongoing			1,200
	Ljubljana 2									Probability sampling	0.9% (late April 2020)	1,368
**Spain**	Barcelona 1							Unknown				7,848
	Barcelona 2	Cohort study						Descriptive cross-sectional		Unknown		10,000
	Madrid					Cohort study				Unknown		45,000
**Sweden**	Stockholm							Unknown				
	Lund					Unknown						
**Turkey**	Istanbul			Descriptive cross-sectional				Cohort; Descriptive cross-sectional				500
	Izmir									Descriptive cross-sectional		3,465
**Total**		**10**		**5**		**8**		**20**		**16**		

**Table 4:** Estimation of the amount of samples used to perform seroprevalance studies, diagnostic serology, to support convalescent plasma studies or other larger studies by participating laboratories

Country	City	Seroprevalance studies	Diagnostic	Convalescent plasma studies	As support for larger studies	Other purposes
**Estimate No. samples**	**Estimate No. samples**	**Estimate No. samples**	**Estimate No. samples**	**Estimate No. samples**
**Austria**	Vienna	>5000	~ 500	~ 500	Multiple evaluation studies for serological assays in acute infection (n ~ 400) national and local seroprevalence studies (n ~ 5000). Correlation of NT with other assays (n ~ 500). Characterization of reconvalescent plasma (n ~ 500)	
**Belgium**	Bruges	-	320	-	-	-
**Bosnia and Herzegovina**	Sarajevo	-	561	-	-	-
**Bulgaria**	Sofia	-	Number not reported	-	-	-
**Croatia**	Zagreb	1050	-	-	-	-
**Czech Republic**	Prague	460	100	50	100	
**Denmark 1**	Copenhagen	-	-	-	-	-
**Denmark 2**	Copenhagen and Hilleroed	~ 10 000	-	-	-	-
**Germany 1**	Kiel		~ 150	-	-	-
**Germany 2**	Freiburg	1000	500	100	Number not reported	-
**Germany 3**	Bonn	>4000	~ 500	~ 120	-	-
**Greece 1**	Thessaloniki	-	-	-	-	To check antibodies kinetics comparing mild and severe cases (70 samples)
**Greece 2**	Crete	2000			Number not reported	-
**Iceland**	Reykjavík		Number not reported	Number not reported	-	-
**Ireland**	Dublin	1733	2285	-	-	-
**Italy**	Pavia	5000	20 000	3900	Correlation of NT with other assays (n ~ 1100) local seroprevalence studies (n ~ 1900). Multiple evaluation studies for serological assays in acute infection (n = ~ 400)	-
**The Netherlands**	Tilburg	~ 140*	Number not reported	-	-	Study on the humoral immune response in mildly and severe COVID-19 patients (62 patients, ~ 180 samples)
**Norway 1**	Oslo	~ 4500	~ 3500	200	Number not reported	-
**Norway 2**	Trondheim		400	150	-	-
**Portugal 1**	Porto	525	-	-	-	-
**Portugal 2**	Mora	250	-	-	-	-
**Portugal 3**	Lisbon 1	-	-	-	-	-
**Portugal 4**	Lisbon 2	2300	-	-	-	-
**Portugal 5**	Porto	60	-	-	-	-
**Portugal 6**	Penafiel	-	2724	-	-	-
**Romania**	Bucharest	do not known	-	-	-	-
**Slovenia 1**	Ljubljana 1	1200	-	-	-	-
**Slovenia 2**	Ljubljana 2	1368	250		3,000	3100 free-market testing
**Spain 1**	Barcelona 1	7848	-	-	-	-
**Spain 2**	Barcelona 2	10 000	1000	-	-	-
**Spain 3**	Madrid	45 000		~ 2000	National seroprevalence study (ENE-COVID) (n ~ 150 000). Clinical trial of convalescent Plasma Therapy (ConPlas-19) (n ~ 2000)	Evaluation of 21 marketed serological assays for determining SARS-CoV-2 IgG or total antibodies ( ~ 100 samples/test)
**Sweden 1**	Stockholm		37 000 during April through June.	Data not available	Data not available	-
**Sweden 2**	Lund	Number not reported	Number not reported	Number not reported	-	-
**Turkey 1**	Istanbul	160	100	400	-	-
**Turkey 2**	Izmir	3465	25	325	-	-
**Turkey 3**	Ankara		1200	50	-	-
**Total number of responses**		**24**	**22**	**13**	**8**	**4**

Within these studies or in diagnostic routine, serological testing was used to confirm a past infection in patients without SARS-CoV-2 diagnosis (26 participants 34), to prove acquired immunity to the infection (n = 15), to confirm past infection in those with a negative diagnosis (n = 15), to estimate the number of asymptomatic infections (n = 13) and to evaluate serological responses in immunocompromised patients (n = 10).

Seroprevalence studies performed used several different designs; the descriptive cross-sectional study design was most commonly used followed by cohort study, convenient or probability sampling (**Table 3**). Targeted sampling was also used. A total of 59 seroprevalence studies were reported as having been performed so far; most of them were focusing on health care workers (HCW, 20 laboratories out of 30), followed by those on general populations (16 laboratories), schools or other institutions (10/30) and blood donors (8/30). Furthermore, 13 HCW studies included health care workers or other personnel such as cleaners, porters, administrative or laboratory staff in hospitals. Other three HCW studies focused on staff members in contact with SARS-CoV-2 infected patients, 2 studies on a specific ward and other 2 on HCW with a previous laboratory confirmed SARS-CoV-2 infection.

Three laboratories reported the observed seroprevalence among HCW; it was 11% in Belgium, 5% in Denmark and 22% in the Netherlands (**Table 3**). Five out of the 16 laboratories performing seroprevalence studies on general population reported a seroprevalence ranging from 0.88% in Slovenia to 5.6% in Belgium and 4 of those laboratories reported also the study population size, which varied from 1368 to 18 000 people involved. In particular, Slovenia appeared to have a significantly lower seroprevalence when compared with other countries reporting higher seroprevalences (0.88% vs 2.2 in Denmark, *P* = 0.001; 0.88% vs 2.9% in Lisbon 2, *P* < 0.0001; 0.88% vs 2.75% in Penafiel, *P* = 0.0001). This difference was also reflected when compared the estimate number of confirmed COVID-19 positive, that was significantly lower in Slovenia (101-1000 cases) compared with Denmark and Portugal that reported >10 000 cases.

Furthermore, 31 laboratories reported the epidemiological information collected with their serological data (**Table 5-8**). Most of them collected sample date (26 laboratories), patient’s age (n = 28), information whether they had a laboratory confirmed SARS-CoV-2 infection and if so, date of diagnosis (n = 26), reported symptoms (n = 22), symptom onset (n = 23) and severity of infection (n = 14) (**Table 5**). Data collected on severity of infection included whether they were hospitalised (n = 17), the length of their hospitals stay (n = 9) or whether they were admitted to the intensive care (n = 14) (**Table 7**). Smaller number of laboratories reported data on outcome of the infection (n = 14, **Table 6**) and the presence of underlining medical conditions (n = 14, **Table 8**). Data on long-term outcomes was rarely collected; data on on-going SARS-CoV-2 PCR positivity was collected by 8 laboratories whereas only a few laboratories systematically collected data on long-term respiratory, cardiac or neurological issues (n = 2, 1 and 1, respectively; **Table 6**, **Table 8**).

**Table 5:** Description of data about patients’ information, collected during seroprevalence studies conducted in Europe, March-June 2020

	Data collected during seroprevalence studies										
**Country**	**Laboratory performing seroprevalence studies**	**Patient information**									
		**Whether laboratory confirmed diagnosis**	**Whether symptomatic**	**Whether hospitalised**	**Which outcome after infection**	**Presence of underlying disease**	**Date of symptoms onset**	**Date of diagnosis**	**Age**	**Date of sample collection**	**Other data/ comments**
**Austria**	Yes	Yes	Yes	Yes	Yes	Yes	Yes	Yes	Yes	Yes	-
**Belgium**	No	Yes	Yes	Yes	Yes	Yes	No	Yes	Yes	Yes	-
**Bosnia and Herzegovina**	No	-	-	-		-	-	-	-	-	-
**Bulgaria**	Yes	Yes	Yes	Yes	Yes	Yes	Yes	Yes	Yes	Yes	-
**Croatia**	Yes	Yes	Yes	Yes	No	Yes	Yes		No	Yes	-
**Czech Republic**	Yes	Yes	Yes	Yes	Yes	Yes	Yes	Yes	Yes	Yes	-
**Denmark 1**	No	-	-	-	-	-	-	-	-	-	-
**Denmark 2**	Yes	Yes	Yes	Yes	Yes	Yes	Yes	Yes	Yes	Yes	Data on 2 HCW studies, but information not available for general population based study
**Germany 1**	-	Yes	-	-	-	-	-	Yes	Yes	Yes	-
**Germany 2**	Yes	Yes	Yes	-	-	Yes	-		Yes	-	-
**Germany 3**	Yes	-	-	-	-	-	-	-	-	-	-
**Greece 1**	No	Yes	Yes	Yes	Yes	Yes	Yes	Yes	Yes	Yes	-
**Greece 2**	Yes	Yes	Yes	Yes		Yes	Yes		Yes	Yes	-
**Iceland**	No	-	-	-	-	-	-	-	-	-	-
**Ireland**	Yes	Yes	Yes	Yes			Yes	Yes	Yes	Yes	-
**Italy**	Yes	Yes	Yes	Yes	Yes	Yes	Yes	Yes	Yes	Yes	-
**The Netherlands**	Yes	Yes	Yes	Yes	Yes	Yes	Yes	Yes	Yes	Yes	-
**Norway 1**	Yes	-	Yes	No	No	No	Yes	Yes	Yes	Yes	-
**Norway 2**	-	-	-	-	-	-	-	-	-	-	-
**Portugal 1**	Yes	No	No	No	No	No	No	Yes	Yes	Yes	Only blood donors
**Portugal 2**	No	Yes	-	Yes	-	-	Yes	Yes	Yes	Yes	Study on residence
**Portugal 3**	No	-	-	-	-	-	-	Yes	Yes	Yes	-
**Portugal 4**	Yes	Yes	Yes	No	-	Yes	Yes	Yes	Yes	Yes	-
**Portugal 5**	Yes	Yes	Yes	Yes	Yes; No	No	Yes	Yes	Yes	Yes	-
**Portugal 6**	Yes	Yes	Yes	Yes	Yes	Yes	Yes	Yes	Yes	Yes	Elderly of nursery homes/residences also studied
**Romania**	Yes	Yes	Yes	Yes	Yes	Yes	Yes	Yes	Yes	Yes	-
**Slovenia 1**	Yes	Yes	Yes	-	-	Yes	Yes	Yes	Yes	Yes	HCW (including nurses and doctors from 2 regional hospitals)
**Slovenia 2**	Yes	Yes	Yes	Yes	-	-	Yes	Yes	Yes	Yes	-
**Spain 1**	Yes	Yes	Yes	Yes	Yes	-	Yes	Yes	Yes	Yes	-
**Spain 2**	Yes	Yes	Yes		-	-	Yes	Yes	Yes	Yes	-
**Spain 3**	Yes	Yes	Yes	Yes	Yes	-	Yes	Yes	Yes	Yes	-
**Sweden 1**	No	-	-	-	-	-	-	-	-	-	-
**Sweden 2**	Yes	Yes	No	No	No	No	Yes	Yes	Yes	Yes	-
**Turkey 1**	Yes	Yes	-	Yes	-	-	-	Yes	Yes	Yes	-
**Turkey 2**	Yes	-	-	-		-	-	-	-	-	No details available at the laboratory
**Turkey 3**	No	-	-	-	-	-	-	-	-	-	-
**Total number of responses**	**25**	**26**	**22**	**21**	**14**	**14**	**23**	**26**	**28**	**27**	**2**

**Table 6:** Description of outcome data collected during seroprevalence studies conducted in Europe, March-June 2020

	Data collected during seroprevalence studies							
**Country**	**Outcome data collected**							
	**Fully recovered**	**Recovered but still RNA positive**	**Death**	**Long term resp issues**	**Long term cardio issues**	**Long term tireness**	**Long term neuro**	**Other outcome/comments**
**Austria**	Yes	Yes	Yes	-	-	-	-	-
**Belgium**	Yes	Yes	Yes	-	-	-	-	-
**Bosnia and Herzegovina**	-	-	-	-	-	-	-	-
**Bulgaria**	-	Yes	Yes	Yes		Yes	-	-
**Croatia**	-	-	-	-	-	-	-	-
**Czech Republic**	Yes	-	-	-	-	-	-	-
**Denmark 1**	-	-	-	-	-	-	-	-
**Denmark 2**	Yes	-	-	-	-	-	-	For the hospital studies only
**Germany 1**	-	-	-	-	-	-	-	-
**Germany 2**	-	-	-	-	-	-	-	-
**Germany 3**	-	-	-	-	-	-	-	-
**Greece 1**	-	-	-	-	-	-	-	-
**Greece 2**	Yes	-	-	-	-	-	-	-
**Iceland**	-	-	-	-	-	-	-	-
**Ireland**	-	-	-	-	-	-	-	-
**Italy**	Yes	Yes	Yes	-	-	-	-	-
**The Netherlands**	Yes	Yes	-	-	-	-	-	-
**Norway 1**	-	-	-	-	-	-	-	-
**Norway 2**	-	-	-	-	-	-	-	-
**Portugal 1**	-	-	-	-	-	-	-	-
**Portugal 2**	-	-	-	-	-	-	-	-
**Portugal 3**	-	-	-	-	-	-	-	-
**Portugal 4**	-	-	-	-	-	-	-	-
**Portugal 5**	Yes	-	-	-	-	-	-	-
**Portugal 6**	Yes	Yes	-	-	-	-	-	-
**Romania**	Yes	-	Yes				-	-
**Slovenia 1**	-	-	-	-	-	-	-	-
**Slovenia 2**	-	-	-	-	-	-	-	-
**Spain 1**	Yes	Yes	-	-	-	-	-	-
**Spain 2**	-	-	-	-	-	-	-	-
**Spain 3**	Yes	Yes	Yes	Yes	Yes	Yes	Yes	ENE-COVID: Data were collected by National Centre for Epidemiology, Institute of Health Carlos III. ConPlas-19: Data were collected by Hospital Universitario Puerta de Hierro-Majadahonda.
**Sweden 1**	-	-	-	-	-	-	-	
**Sweden 2**	-	-	-	-	-	-	-	Data not collected
**Turkey 1**	Yes		Yes		-	-	-	
**Turkey 2**	-	-	-	-	-	-	-	No details available at the laboratory
**Turkey 3**	-	-	-	-	-	-	-	
**Total number of responses**	**12**	**8**	**9**	**2**	**1**	**2**	**1**	**2**

**Table 7:** Description of hospitalisation data collected during seroprevalence studies conducted in Europe, March-June 2020

	Data collected during seroprevalence studies					
**Country**	**Hospitalisation data collect**					
	**Hospitalisation (non ICU)**	**Not hospitalised**	**Admission in ICU**	**Length of hospitalisation**	**Not known**	**Other**
**Austria**	Yes	Yes	Yes	Yes	-	-
**Belgium**	Yes	Yes	Yes	Yes	-	-
**Bosnia and Herzegovina**	-	-	-	-	-	-
**Bulgaria**	Yes	Yes	Yes	-	-	-
**Croatia**	Yes	Yes	-	-	-	Only data about hospitalization (Y/N)
**Czech Republic**	-	Yes	-	-	-	
**Denmark 1**	-	-	-	-	-	-
**Denmark 2**	Yes	Yes	Yes	Yes	-	For the hospital studies only
**Germany 1**	-	Yes	-	-	-	-
**Germany 2**	Yes	Yes	Yes	Yes	-	-
**Germany 3**				-	-	-
**Greece 1**	Yes	Yes	Yes	-	Yes	-
**Greece 2**	Yes	Yes	Yes	-	-	-
**Iceland**	-	-	-	-	-	-
**Ireland**	-	-	-	-	-	-
**Italy**	Yes	-	Yes	Yes	-	-
**The Netherlands**	Yes	Yes	-	-	-	-
**Norway 1**	-	-	-	-	-	-
**Norway 2**	-	-	-	-	-	-
**Portugal 1**	-	-	-	-	-	-
**Portugal 2**	-	-	-	-	-	-
**Portugal 3**	Yes	-	Yes	-	-	-
**Portugal 4**	-	-	-	-	-	-
**Portugal 5**	Yes	Yes	-	-	-	-
**Portugal 6**	-	-	-	-	-	-
**Romania**	Yes	Yes	Yes	Yes	-	-
**Slovenia 1**	-	-	-	-	-	-
**Slovenia 2**	Yes	Yes	Yes	Yes	-	-
**Spain 1**	-	-	-	-	-	-
**Spain 2**	Yes	Yes	Yes	Yes	-	-
**Spain 3**	Yes	Yes	Yes	Yes	Yes	ENE-COVID: Data were collected by National Centre for Epidemiology, Institute of Health Carlos III. ConPlas-19: Data were collected by Hospital Universitario Puerta de Hierro-Majadahonda.
**Sweden 1**	-	-	-	-	-	-
**Sweden 2**	-	-	-	-	-	-
**Turkey 1**	Yes	Yes	Yes	-	-	-
**Turkey 2**	-	Yes	-	-	-	-
**Turkey 3**	-		-	-	-	-
**Total number of responses**	**17**	**18**	**14**	**9**	**2**	**3**

**Table 8:** Description of risk factors data collected and age-groups included in seroprevalence studies conducted in Europe, March-June 2020

Country	Risks data collected									Age-group (years) included in the study/ies						
**Hypertension**	**Diabetes**	**Cardiovascular disease**	**Chronic respiratory**	**Chronic kidney**	**Immune compromised**	**Cancer**	**Obesity**	**Other**	**0-4**	**5-9**	**10-19**	**20-59**	**60-74**	**75-90**	**>90**	
**Austria**	Yes	Yes	Yes	Yes	Yes	Yes	Yes	Yes	-	-	Yes	Yes	Yes	Yes	Yes	Yes	
**Belgium**	-	-	-	-	Yes	-	-	-	HCW	-	-	-	Yes	Yes	Yes	Yes	
**Bosnia and Herzegovina**	-	-	-	-	-	-	-	-	-	-	-	-	-	-	-	-	
**Bulgaria**	-	-	-	-	-	Yes	-	-	Haematological	-	-	-	Yes	Yes	Yes	-	
**Croatia**	-	-	-	-	-	-	-	-	Data not systematically collect	Yes	Yes	Yes	Yes	Yes	Yes	-	
**Czech Republic**	-	-	-	-	-	-	-	-	No criteria of inclusion	-			Yes	Yes	Yes	-	
**Denmark 1**	-	-	-	-	-	-	-	-	-	-	-	-	-	-	-	-	
**Denmark 2**	Yes	Yes	Yes	Yes	Yes	Yes	Yes	Yes	For the hospital studies only	-	-	Yes	Yes	Yes	Yes	Yes	
**Germany 1**	-	-	-	-	-	-	-	-	-	-	-	Yes	Yes	Yes	Yes	-	
**Germany 2**	-	-	-	-	-	-	-	-	-	-	Yes	Yes	Yes	Yes	Yes	-	
**Germany 3**	-	-	-	-	-	-	-	-	-	-	-	Yes	Yes	Yes	Yes	-	
**Greece 1**	-	-	-	-	-	-	-	-	-	-	-	-	Yes	Yes	Yes	-	
**Greece 2**	-	-	-	-	-	-	-	-	-	-	-	-	Yes	Yes	Yes	-	
**Iceland**	-	-	-	-	-	-	-	-	-	-	-	-	-	-	-	-	
**Ireland**	-	-	-	-	-	-	-	-	-	-	-	-	-	-	-	-	
**Italy**	-	-	-	-	-	-	-	-	-	-	-	-	Yes	Yes	Yes	-	
**The Netherlands**	-	-	Yes	Yes	-	Yes	Yes	Yes	Questionnaire about risk factors	-	-	.	Yes	Yes	-	.	
**Norway 1**	-	-	-	-	-	-	-	-	-	-	Yes	Yes	Yes	Yes	Yes	-	
**Norway 2**	-	-	-	-	-	-	-	-	-	-	-	-	-	-	-	-	
**Portugal 1**	-	-	-	-	-	-	-	-	-	-	-	Yes	Yes	Yes	-	-	
**Portugal 2**	-	-	-	-	-	-	-	-	-	-	-	-	-	-	-	-	
**Portugal 3**	-	-	-	-	-	-	-	-	-	Yes	Yes	Yes	-	-	-	-	
**Portugal 4**	Yes	Yes	Yes	Yes	Yes	Yes	Yes	Yes	-	Yes	Yes	Yes	Yes	Yes	Yes	Yes	
**Portugal 5**	-	-	-	-	-	-	-	-	HCW with previously positive real time PCR were included	-	-	-	Yes	Yes	-	-	
**Portugal 6**	Yes	Yes	Yes	Yes	Yes	-	Yes	-	Questionnaires about risk factors were performed. Elderly from nursery homes/residences were also considered	Yes	Yes	Yes	Yes	Yes	Yes	Yes	
**Romania**	Yes	Yes	Yes	Yes	Yes	Yes	Yes	Yes	-	-	-	-	Yes	Yes	-	-	
**Slovenia 1**	Yes	Yes	Yes	Yes	Yes	Yes	Yes	-	HCW	-	-	-	Yes	Yes	-	-	
**Slovenia 2**	-	-	-	-	-	-	-	-	-	Yes	Yes	Yes	Yes	Yes	Yes	Yes	
**Spain 1**	-	-	-	-	-	-	-	-	-	-	-	-	Yes	-	-	-	
**Spain 2**	-	-	-	-	-	-	-	-	-	Yes	Yes	Yes	Yes	Yes	Yes	Yes	
**Spain 3**	Yes	Yes	Yes	Yes	Yes	Yes	Yes	Yes	ENE-COVID: Data are collected by National Centre for Epidemiology, Institute of Health Carlos III. ConPlas-19: Data are collected by Hospital Universitario Puerta de Hierro-Majadahonda.	Yes	Yes	Yes	Yes	Yes	Yes	Yes	
**Sweden 1**	-	-	-	-	-	-	-	-	-	-	-	-	-	-	-	-	
**Sweden 2**	-	-	-	-	-	-	-	-	Data not systematically collect	Yes	Yes	Yes	Yes	Yes	Yes	Yes	
**Turkey 1**	-	-	-	-	-	-	-	-	-	-	-	-	Yes	-	-	-	
**Turkey 2**	-	-	-	-	-	-	-	-	No details available at the laboratory	-	-	-	-	-	-	-	
**Turkey 3**	-	-	-	-	-	-	-	-	-	-	-	-	-	-	-	-	
**Total number of responses**	**6**	**6**	**8**	**8**	**7**	**8**	**8**	**6**	**10**	**8**	**11**	**14**	**26**	**24**	**19**	**9**	

## DISCUSSION

This study reports data on SARS-CoV-2 serological testing from 36 clinical or public health virological laboratories in 20 European countries. Results demonstrate a rapid European-wide hospital-based response to the COVID-19 pandemic we all faced earlier this year. While these largely hospital-based laboratories were diagnosing and controlling over 100.000 patients with SARS-CoV-2 infection based on defined criteria [6], followed by a variety of mitigation and quarantine strategies often affecting also themselves, they managed simultaneously to introduce a battery of different serological assays not only to clinical practice but also to support seroprevalence studies. Our data shows the variety of assays introduced was vast; from viral isolation to in-house testing and commercial assays, containing different antigens and measuring different antibody classes.

However, during the first wave of COVID-19 pandemic, mitigation strategies were broadly applied without strong evidence to support these [8]. All countries and laboratories participating in our study experienced these mitigation strategies including national and local lockdowns and closures of restaurants, schools or other public places. Measures reflected also the health system capacity, such as observed in Bulgaria where the national lockdown lasted throughout the study period despite the small number of reported SARS-CoV-2 cases. On the contrary, Sweden with over 15 000 SARS-CoV-2 infections, choose to recommend social distancing only including limiting the gatherings to maximum 50 people, working from home and restricting access to care-homes, in line with the ECDC guidance [13]. Along with mitigation strategies, most participating laboratories (94%, 34/36) reported also the use of quarantine scheme after a suspected contact with a SARS-CoV-2 positive person, a travel within or beyond the Europe, with the majority of responders indicating 14 days as period of observation as recommended by ECDC [14]. The length of quarantine was also in keeping with suggested SARS-CoV-2 incubation period [15,16]. It is clear from our data that about 80% of regions/countries observed a decrease in the number of reported SARS-CoV-2 cases within the first 60 days of national lockdown.

This pandemic has also demonstrated the fast tempo serological assays are needed, and can be developed, to support investigations into a newly emerged virus, such as SARS-CoV-2 here. To date, 10 months from the first diagnosis in Europe, about 200 commercial assays have been introduced into the European market [17] and the number continues to increase. Although the clinical and research needs for such assays cannot be over-stated, it is important to maintain quality standards in clinical laboratories. For these reasons, we need to consider how the international community can reach out and support the diagnostic laboratories during these challenging times. In our series of laboratories, validation or verification of the assays prior to their introduction into routine work was largely undertaken. However, the number of samples used for the validation was limited in some cases (ie, below 15 in total) and some laboratories did not have access to reference material in order to perform the assay validation. However, their responses indicated understanding of general good laboratory practice. In fact, proper validation prior the use of a new commercial assay should be always performed, but as openly declared from one of participant, during the first pandemic wave laboratories were overwhelmed due to the amount of work requested and hence had to postpone the validation for practical reasons. The guidance on assay validation is limited, but it should also be modified according to specific situation such as pandemic. Furthermore, informal hospital-based laboratory networks should be encouraged as they can be a source of technical and material support during difficult times, like this pandemic.

Although we did not aim to evaluate the results obtained by different serological assays in this study, it is vital to point out that the sensitivity and specificity of available SARS-CoV-2 serological assays varies hugely [18]. For example, the sensitivity of some most commonly used assays does not reach even 80% [19]. However, serological assays have different requirement depending on their purpose. Whereas the ease of use might be the most important criteria if an assay is being used for a large-scale seroprevalence work, sensitivity is a key marker for any assay used diagnostically. Any assay used to screen convalescent plasma donors should be predictive of high neutralising antibody levels. Anticipated SARS-CoV-2 vaccine introduction into routine use will set up further requirements for serological assays used, especially when we need to evaluate separately vaccine-produced and natural immunity. This would mean that laboratories may need to consider setting up at least two serological assays, one focusing on antibodies directed against spike protein and another against nucleocapsid proteins. Based on our data, over half of laboratories participating to our study do not currently have both of these assays available, but by introducing an additional test they could easily support the introduction of vaccine.

More than half of participating laboratories performed one or more seroprevalance studies, mostly focusing on specific population such as health care workers and blood donors. With these studies, information on age, date of sample collection and diagnosis were almost always gathered, whereas the outcome data and information on underlying diseases were less frequently collected. [20]. This could be a limitation in understanding risk factors driving a different immunological response. In fact, despite it is quite clear that the presence of some diseases can increase the risk of severe outcomes [18], their role in the immune response remain unclear [21]. Although our current understanding of immunological responses to SARS-CoV-2 including the durability of the antibody response and occurrence of seroreversion remains uncertain [22,23], continuation of seroprevalance studies collecting more information of medical conditions is highly recommended. Such studies will provide better understanding of the pandemic course in the coming months in the absence of a protective vaccine and the necessity to target public health measures to specific populations at highest risk.

Estimation of seroprevalance in general population was reported by five participant laboratories in this study, and similarly seroprevalence among HCW was reported by three participant laboratories only. Although it was clear, that seroprevalence was higher among the HCW than in general population, confirming the previous observations [24], it is not possible to draw further conclusions from these data. We collected only limited information about the characteristics of the study population (ie, age, symptoms), details about the serological test used (ie, manufacturer, antigen and antibody subclass) and the dates of study. Although the same limitations applied to the population-based seroprevalance studies reported here, the seroprevalence estimates were in line with the number of SARS-CoV-2 infections reported during the same period from the same region hence reflecting the country-based situation well.

We have described the SARS-CoV-2 serological testing introduced and undertaken across the 36 different European laboratories during the first pandemic wave, when there was the urge to understand the seroprevalance of affected populations, the length of immunity acquired during SARS-CoV-2 infections and its likely protective effects. This understanding is still required and would likely support the public health decision making and drive evidence-based mitigation and infection control strategies.

## CONCLUSION

This is the first paper, to our knowledge, gathering the information available in the European region about the use of serological testing during the first pandemic wave. The study documents the high level of hospital-laboratory involvement in serological response across European countries during the first pandemic wave: serology methods were rapidly implemented by many hospital laboratories and served as an important diagnostic supplement while the molecular diagnostic capacity increased during these early months of the first pandemic wave.

**Note added in proof:** The probability-based seroprevalence study in Slovenia (Table 3, Ljubljana 2) is now published [25].
